# Supplementary material for: Multi-Locus Phylogenetic Analysis Revealed the Association of Six Colletotrichum Species with Anthracnose Disease of Coffee (Coffea arabica L.) in Saudi Arabia
Source: J Fungi (Basel). 2023 Jun 27;9(7):705. doi: 10.3390/jof9070705 (PMC10381574; doi:10.3390/jof9070705)
Supplement: Supplementary file 1 [file jof-09-00705-s001.zip › jof-2452665-supplementary.pdf]

Table S1. Source, origin and date of collection of the 27 isolates obtained in this study

Isolate Number	Identity	Collection date	Isolation source	Host	Location
PPDU26A	*C. siamense*	13/10/2022	leaves	*Coffea arabica*	Jazan, Saudi Arabia
PPDU26B	*C. coffeae-arabicae*	13/10/2022	leaves	*Coffea arabica*	Jazan, Saudi Arabia
PPDU27B	*C. siamense*	13/10/2022	branch	*Coffea arabica*	Jazan, Saudi Arabia
PPDU27D	*C. coffeae-arabicae*	13/10/2022	branch	*Coffea arabica*	Jazan, Saudi Arabia
PPDU27M	*C. siamense*	13/10/2022	fruit	*Coffea arabica*	Jazan, Saudi Arabia
PPDU28A	*C. aeschynomenes*	13/10/2022	fruit	*Coffea arabica*	Jazan, Saudi Arabia
PPDU28C	*C. saudianum*	13/10/2022	twig	*Coffea arabica*	Jazan, Saudi Arabia
PPDU28E	*C. saudianum*	13/10/2022	fruit	*Coffea arabica*	Jazan, Saudi Arabia
PPDU28L	*C. saudianum*	13/10/2022	twig	*Coffea arabica*	Jazan, Saudi Arabia
PPDU28J	*C. saudianum*	14/10/2022	leaves	*Coffea arabica*	Jazan, Saudi Arabia
PPDU29A	*C. saudianum*	14/10/2022	leaves	*Coffea arabica*	Jazan, Saudi Arabia
PPDU29B	*C. saudianum*	14/10/2022	leaves	*Coffea arabica*	Jazan, Saudi Arabia
PPDU29H	*C. siamense*	14/10/2022	leaves	*Coffea arabica*	Jazan, Saudi Arabia
PPDU29F	*C. coffeae-arabicae*	12/9/2022	leaves	*Coffea arabica*	Jazan, Saudi Arabia
PPDU31I	*C. saudianum*	12/9/2022	leaves	*Coffea arabica*	Al Baha, Saudi Arabia
PPDU31M	*C. saudianum*	12/9/2022	leaves	*Coffea arabica*	Al Baha, Saudi Arabia
PPDU32A	*C. coffeae-arabicae*	12/9/2022	leaves	*Coffea arabica*	Al Baha, Saudi Arabia
PPDU32B	*C. siamense*	12/9/2022	leaves	*Coffea arabica*	Al Baha, Saudi Arabia
PPDU36S	*C. phyllanthi*	15/11/2022	leaves	*Coffea arabica*	Asir, Saudi Arabia
PPDU38I	*C. saudianum*	15/11/2022	leaves	*Coffea arabica*	Asir, Saudi Arabia
PPDU38B	*C. saudianum*	15/11/2022	leaves	*Coffea arabica*	Asir, Saudi Arabia
PPDU38H	*C. saudianum*	15/11/2022	leaves	*Coffea arabica*	Asir, Saudi Arabia
PPDU38F	*C. saudianum*	15/11/2022	leaves	*Coffea arabica*	Asir, Saudi Arabia
PPDU39E	*C. siamense*	15/11/2022	branch	*Coffea arabica*	Asir, Saudi Arabia
PPDU39D	*C. siamense*	15/11/2022	branch	*Coffea arabica*	Asir, Saudi Arabia
PPDU40G	*C. siamense*	13/12/2022	leaves	*Coffea arabica*	Asir, Saudi Arabia
PPDU41K	*C. karstii*	13/12/2022	leaves	*Coffea arabica*	Asir, Saudi Arabia
